# Resting-state EEG microstates as electrophysiological biomarkers in post-stroke disorder of consciousness

**DOI:** 10.3389/fnins.2023.1257511

**Published:** 2023-10-02

**Authors:** Fang Yu, Yanzhe Gao, Fenglian Li, Xueying Zhang, Fengyun Hu, Wenhui Jia, Xiaohui Li

**Affiliations:** ^1^College of Electronic Information and Optical Engineering, Taiyuan University of Technology, Taiyuan, China; ^2^College of Life Sciences, Nankai University, Tianjin, China; ^3^The Fifth Clinical Medical College of Shanxi Medical University, Department of Neurology, Shanxi Provincial People’s Hospital, Taiyuan, China

**Keywords:** microstates, disorder of consciousness, EEG, post-stroke, biomarkers

## Abstract

**Introduction:**

Ischemic stroke patients commonly experience disorder of consciousness (DOC), leading to poorer discharge outcomes and higher mortality risks. Therefore, the identification of applicable electrophysiological biomarkers is crucial for the rapid diagnosis and evaluation of post-stroke disorder of consciousness (PS-DOC), while providing supportive evidence for cerebral neurology.

**Methods:**

In our study, we conduct microstate analysis on resting-state electroencephalography (EEG) of 28 post-stroke patients with awake consciousness and 28 patients with PS-DOC, calculating the temporal features of microstates. Furthermore, we extract the Lempel-Ziv complexity of microstate sequences and the delta/alpha power ratio of EEG on spectral. Statistical analysis is performed to examine the distinctions in features between the two groups, followed by inputting the distinctive features into a support vector machine for the classification of PS-DOC.

**Results:**

Both groups obtain four optimal topographies of EEG microstates, but notable distinctions are observed in microstate C. Within the PS-DOC group, there is a significant increase in the mean duration and coverage of microstates B and C, whereas microstate D displays a contrasting trend. Additionally, noteworthy variations are found in the delta/alpha ratio and Lempel-Ziv complexity between the two groups. The integration of the delta/alpha ratio with microstates’ temporal and Lempel-Ziv complexity features demonstrates the highest performance in the classifier (Accuracy = 91.07%).

**Discussion:**

Our results suggest that EEG microstates can provide insights into the abnormal brain network dynamics in DOC patients post-stroke. Integrating the temporal and Lempel-Ziv complexity microstate features with spectral features offers a deeper understanding of the neuro mechanisms underlying brain damage in patients with DOC, holding promise as effective electrophysiological biomarkers for diagnosing PS-DOC.

## 1. Introduction

Stroke is widely acknowledged as the second leading cause of death and a prominent contributor to disability globally (Feigin et al., 2017, 2021), which results in severe behavioral impairments and widespread structural and functional network disruptions (Alkhachroum et al., 2022). Post-stroke, patients commonly experience symptoms such as disorder of consciousness (DOC) or coma, which contribute to increasing in-hospital mortality and unfavorable outcomes upon discharge for stroke patients (Li et al., 2016). Therefore, it is crucial to diagnose post-stroke disorder of consciousness (PS-DOC) promptly and accurately, while gaining a comprehensive understanding of the neural mechanisms underlying brain injury. Traditionally, clinical rating scales like the Glasgow Coma Scale (GCS) and Coma Recovery Scale Revision (CRS-R) have been used to assess patients with DOC. Although clinical behavioral assessment remains the gold standard (Hermann et al., 2020), these scoring systems exhibit high inter-rater and inter-examiner variability and lack objective evidence of central nervous system damage following brain injury (Claassen et al., 2016; Giacino et al., 2018; Song et al., 2018).

Currently, the utilization of electrophysiological methods, specifically electroencephalography (EEG), to measure neurological function in patients has been demonstrated as an effective method for rapidly assisting in the diagnosis of DOC (Bai et al., 2021b; Ballanti et al., 2022; Duszyk-Bogorodzka et al., 2022). Extensive research utilizing EEG-based spectral analysis, source imaging analysis, and graph theory analysis has improved neurophysiological assessments in the fields of stroke and DOC rehabilitation and diagnosis (Finnigan et al., 2016; Bai et al., 2021a; Zhuang et al., 2022; Bouchereau et al., 2023; Colombo et al., 2023). Regarding spectral patterns, previous studies have documented a notable reduction in alpha power after brain injury, including stroke (Edlow et al., 2021). Consequently, stroke leads to a marked elevation in the delta/alpha ratio (DAR), which quantifies the ratio of delta band power to alpha band power (Finnigan and van Putten, 2013). Likewise, distinguishing between patients with DOC and healthy controls often relies on the analysis of delta and alpha frequency bands, where increased delta rhythms and diminished alpha rhythms serve as prominent indicators of reduced consciousness levels (Rossi Sebastiano et al., 2015). Specifically, DOC patients demonstrate higher delta power than healthy controls (Sitt et al., 2014), while an augmentation in alpha power is observed during the recovery of consciousness in these individuals (Stefan et al., 2018). These studies seem to suggest that we can observe the relationship between the spectral feature DAR and reduced consciousness in patients with PS-DOC.

Although traditional spectral analysis of resting-state EEG integrates brain activity over several seconds in different frequency bands, this method fails to capture the spatial and temporal characteristics of resting-state brain networks occurring at shorter time scales (e.g., tens of milliseconds; Li et al., 2022). In contrast, multi-channel fusion of EEG microstate analysis can capture the spatiotemporal dynamics of activity in different brain regions at a sub-second time scale (Bréchet et al., 2019). Microstates represent specific topological patterns of electrical potentials and are typically classified into four distinct classes (Michel and Koenig, 2018), and the microstates persist for a transient period of approximately 60–120 ms in a quasi-stable state before rapidly transitioning to another microstate category (Lehmann et al., 1987). The swift transitions between microstates reflect rapid changes in brain dynamics, revealing the interconnectedness between cognitive function, information processing, and neural regulation in the brain (Khanna et al., 2014; Von Wegner et al., 2018; Liu et al., 2020). Furthermore, different microstate classes exhibit strong associations with specific resting-state networks (RSNs) in the brain, including the auditory network, visual network, salience network, and attention network, among others (Britz et al., 2010; Michel and Koenig, 2018). Increasing evidence suggests that abnormal alterations in temporal characteristics (such as mean duration, coverage, and occurrence) of microstates are observed in various neuropsychiatric disorders, including post-traumatic stress disorder (Terpou et al., 2022), schizophrenia (Rieger et al., 2016; Lin et al., 2022), Alzheimer’s disease (Tait et al., 2020), Parkinson’s disease (Pal et al., 2021), and depression (Zhao et al., 2022). However, microstate analysis research related to DOC primarily focuses on patients with diverse etiologies, including brain trauma, intracranial bleeding, hypoxic–ischemic, and other conditions (Guo et al., 2022; Toplutaş et al., 2023; Zhang et al., 2023). In contrast, there is limited research on microstate analysis in DOC patients with a single etiology, such as ischemic stroke, and our understanding of the temporal dynamics and spatiotemporal interaction effects in their brains remains insufficient.

Moreover, substantial evidence suggests that microstate time sequences display dynamic and nonlinear characteristics, including non-Markovian transition behaviors, where the transition to the next microstate class is independent of the current microstate class (Gschwind et al., 2015; Von Wegner et al., 2017). Increasing studies have introduced nonlinear measures applied to microstate sequences. In particular, Tait et al. pioneered the utilization of the Lempel-Ziv complexity (LZC) algorithm to investigate microstate transition patterns (Tait et al., 2020), revealing a reduction in microstate LZC among individuals with Alzheimer’s disease in comparison to their healthy counterparts. Subsequently, Zhang et al. explored the alterations in the LZC of microstate sequences in patients with brain diseases (Zhang et al., 2021), and Zhao et al. discovered an increase in the LZC of microstates in adolescents with depression (Zhao et al., 2022). Nonlinear analysis of EEG microstate sequences quantifies the persistent characteristics of brain electrical activity, revealing complex dynamic changes at very small time scales (Von Wegner et al., 2023). We suggest that the microstate LZC in PS-DOC patients may also exhibit some degree of abnormality, providing new insights into the neuro anomalies associated with DOC.

The aforementioned analysis indicates that the current understanding of EEG microstates in PS-DOC remains limited. To this end, the innovations and contributions of our study are summarized as follows.

Firstly, to the best of our knowledge, this paper is the first work to investigate the differences in EEG microstates between PS-DOC patients and post-stroke awake (PS-AW) conscious state patients. Comparison results show that there exist differences in microstate topographies between the two groups and especially exhibit significant alterations in temporal features among them.

Secondly, we analyze the Lempel-Ziv complexity of the microstate time sequences and find that, there exhibits higher repetitiveness and slower transition trends in the microstates of PS-DOC patients than that of PS-AW patients. Additionally, to supplement the spectral information in resting-state EEG, we calculate the DAR of spectral features in both groups. We find that DAR is significantly higher in PS-DOC patients.

Finally, we explore the potential of the aforementioned extracted features that are sensitive to intergroup variability in the classification of DOC. In particular, we fuse these features and feed the combined sets into an SVM classifier to identify the DOC among stroke patients. The outcome demonstrates that our work could accurately identify 92.86% of DOC patients.

In summary, our study contributes to a better understanding of resting-state EEG microstate features in patients with DOC post-stroke, helps us to identify potentially valid electrophysiological biomarkers, and provides important insights and neurological evidence into the causative mechanisms of decreased levels of consciousness post-stroke.

## 2. Materials and methods

### 2.1. Patients

We retrospectively investigate 60 patients diagnosed with ischemic stroke who received treatment between June 2020 and January 2022 at the stroke center of Shanxi Provincial People’s Hospital. The inclusion criteria include (1) First-time diagnosis of ischemic stroke; (2) Subacute stroke (2 weeks to 3 months post-stroke); (3) Complete assessment of consciousness and scalp EEG measurement; (4) No other neurological disorders. Exclusion criteria encompass (1) Patients aged below 18; (2) Pregnant or lactating women; (3) Patients with traumatic brain injury or intracerebral hemorrhage; (4) Patients who underwent thrombolysis surgery. Two patients are excluded due to other neurological comorbidities, and two patients are excluded as a result of receiving thrombolytic surgery. Finally, we obtain a sample of 56 patients with ischemic stroke (mean age = 67.09 years, 31 males, 25 females, and 56 right-handed individuals).

The consciousness state of the patients is assessed using the GCS by clinical experts in neurology, who are blinded to the patient’s EEG measurements. Based on the GCS scores, we divide the patients into two groups: 28 patients with post-stroke awake consciousness (PS-AW; GCS > 13; mean age = 68.96 years, 17 males, 11 females), representing stroke patients without DOC; and 28 patients with post-stroke DOC (PS-DOC; GCS < 13; mean age = 65.21 years, 14 males, 14 females), including 13 patients with moderate DOC (12 > GCS > 9) and 15 patients with severe DOC/coma (3 < GCS < 8). Furthermore, the *t*-tests are performed for age and post-stroke onset time between the two groups and reveal no noticeable distinctions (*t* = −1.08, *p* = 0.285; *t* = 1.49, *p* = 0.14). The chi-square test for gender distribution also yields similar results (*χ*^2^ = 0.48, *p* = 0.49). All EEG data collection followed a retrospective observational cohort study which was approved by the local institutional review board (Ethics Committee of Shanxi Provincial People’s Hospital). The demographic and clinical information of all patients is summarized in Table 1.

**Table 1:** Patients’ demographic and clinical information.

	PS-AW patients (*n* = 28) (Mean ± SD)	PS-DOC patients (*n* = 28) (Mean ± SD)		Group comparison
Age, years	68.96 ± 11.63	65.21 ± 14.21		*t*(54) = −1.08, *p* = 0.29
Gender (Females/Males)	11/17	14/14		*χ*^2^ = 0.48, *p* = 0.49
Weeks post-stroke	7.82 ± 2.91	6.64 ± 3.01		*t*(54) = 1.49, *p* = 0.14
GCS	>13	9 < *n* = 13 < 12	3 < *n* = 15 < 8	
Consciousness state	Awake	Moderate DOC	Severe DOC/Coma	

SD, standard deviation. Group comparison: The chi-square test for gender and t-test for age and weeks after stroke are performed between PS-AW and PS-DOC patients, there are no statistically significant differences observed.

### 2.2. Electroencephalography data acquisition and preprocessing

Each patient is instructed to lie supine on the bed with their eyes closed within 30 min after the clinical consciousness assessment. Resting-state EEG data are collected using a bedside monitoring system (Solar 2000 N, Solar Electronic Technology Co., Ltd., Beijing, China) with a sampling rate of 100 Hz, for a minimum duration of 10 min. 19 Ag/AgCl ring electrodes (FP1, FP2, F3, F4, C3, C4, P3, P4, O1, O2, F7, F8, T3, T4, T5, T6, FZ, CZ, and PZ) are positioned on the scalp according to the international 10–20 system (Jurcak et al., 2007), ensuring the impedance of all electrodes remained below 10 kΩ. The right mastoid serves as the reference electrode, while the forehead is designated as the location for the ground electrode. Additionally, a pair of electrooculography electrodes are also employed to capture eye movements and blinking patterns.

**Figure 1:**
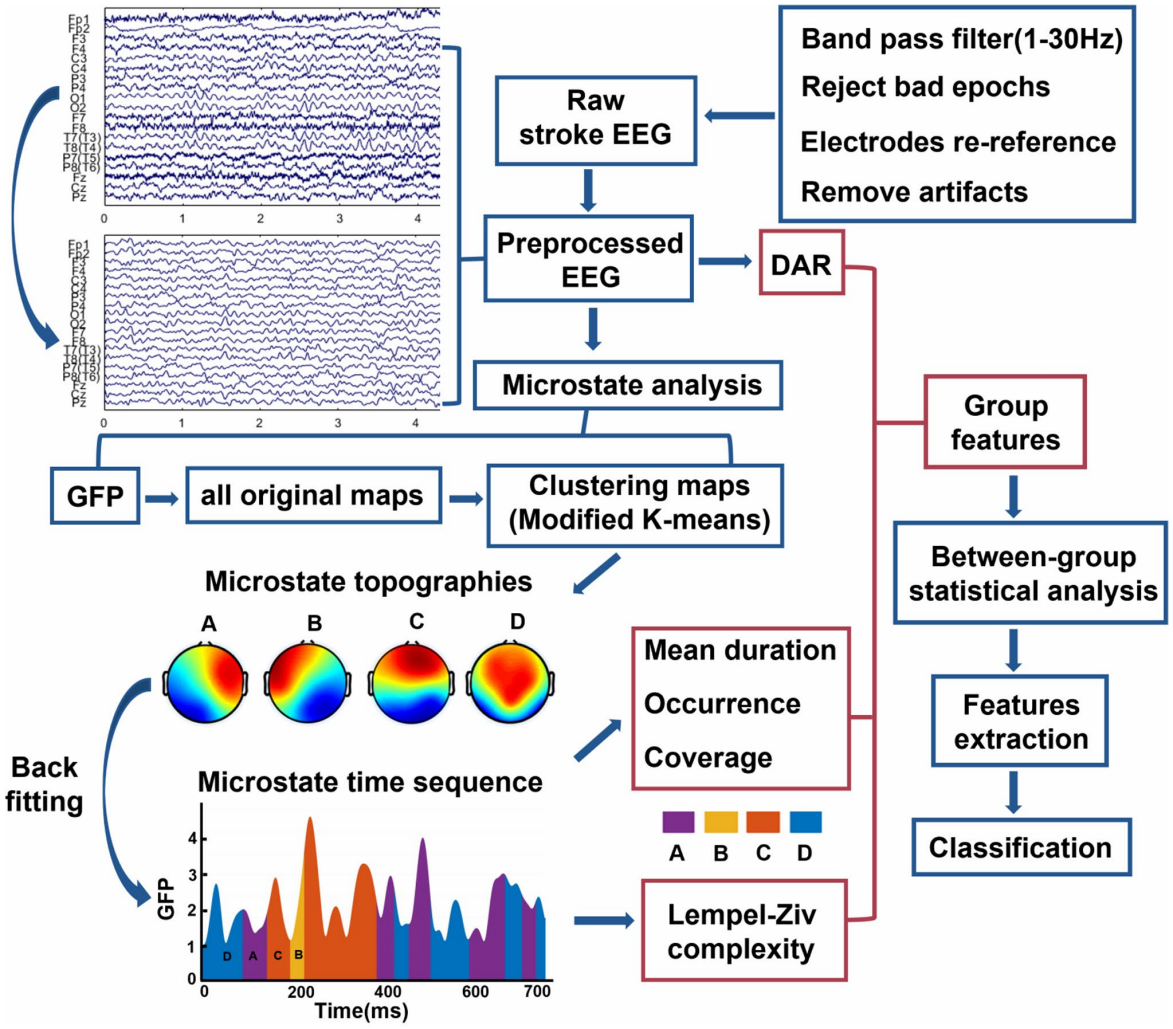
Overview of analytical methods for this study. The contents of red boxes are the three types of group features in the study, see more details in “Materials and methods” section. DAR, delta/alpha ratio; GFP, global field power.

The offline preprocessing of raw EEG data is conducted using MATLAB (R2019a, MathWorks Inc., United States) in conjunction with the EEGLAB 2021.0 toolbox (Delorme and Makeig, 2004). The general preprocessing steps can be seen in Figure 1. Specifically, we utilize a finite impulse response filter for bandpass filtering in the frequency range of 1 Hz to 30 Hz, along with a notch filter designed to eliminate power line interference at 50 Hz. We conduct visual inspection to remove artifacts related to eye movements or blinking that are highly correlated with electrooculography signals, followed by re-referencing the EEG data using the common average reference. Subsequently, applying the *runica* algorithm from the EEGLAB, we perform independent component analysis on the EEG data, and the ICLabel and ADJUST algorithms from the plugin extension are employed to remove noise related to muscle activity, and eye movements. The count of removed artifact-independent components in the PS-AW group and the PS-DOC group are 2.3 ± 0.7 (mean ± standard deviation) and 1.8 ± 0.3 (mean ± standard deviation), respectively, with no significant difference. After EEG preprocessing, each patient’s artifact-free epochs of 60 s are retained for data analysis.

### 2.3. Delta/alpha ratio calculation

In the spectral analysis of the preprocessed EEG, we utilize the Welch method to compute the average power within the 1–30 Hz frequency range for each electrode. To segment the signal, a Hanning window with a duration of 2.048 s is applied, using a 50% window overlap and a frequency resolution of 0.488 Hz approximately. The power spectral density within each time window is then calculated using the Fast Fourier Transform, resulting in power values within the frequency range of 1–30 Hz. These power values are summed for each window to obtain the total power within that window. Subsequently, the average total power across all windows is computed to determine the average power. For each electrode, the average power spectrum is derived by averaging the results from all 19 electrodes, resulting in the “average scalp power spectrum” (Finnigan et al., 2007). Finally, the ratio of the average power spectrum is calculated over the specific frequency range of interest, including the delta band (1–4 Hz) and the alpha band (8–13 Hz), yielding the EEG spectral characteristic known as the DAR. It is worth noting that although we focus on extracting the power spectra for the delta and alpha bands here, we retain the broader frequency range of 1–30 Hz for subsequent microstate analysis.

### 2.4. Microstates analysis

We perform microstate analysis on preprocessed EEG data using the Microstate EEGLAB Toolbox (Poulsen et al., 2018). The analysis flow is illustrated in Figure 1. Initially, the global field power (GFP) is extracted from the EEG signal in both stroke groups. GFP is defined as follows:


$$GFP=\sqrt{\frac{\sum_{i}^{n}{\left[u_{i}\left(t\right)-u_{\mathrm{mean}}\left(t\right)\right]}^{2}}{n}}$$

Where $u_{i}\left(t\right)$ is the instantaneous potential of the *i*th electrode at time *t*, $u_{\mathrm{mean}}\left(t\right)$ is the average instantaneous potential across all electrodes at time *t*, and n denotes the number of electrodes ( n is 19 in our study). GFP is calculated as the standard deviation of the EEG signals from all electrodes (Murray et al., 2008). Considering that the local maximum (peak) of GFP exhibits a favorable signal-to-noise ratio and stable changes in EEG topography, we set a minimum interval of 10 ms between consecutive GFP peaks. We extract the brain topographic maps (also known as original maps) at 2000 randomly selected GFP peak locations. GFP peaks that exceed twice the standard deviation are also removed, as they often contain artifacts of non-neuronal origin. Subsequently, all obtained original maps are input into a modified k-means clustering algorithm (ignoring polarity). We employ an optimized segmentation scheme with 1,000 iterations to obtain the best clustering. In line with previous studies (Zappasodi et al., 2017), we identify the optimal number of prototype microstates as 4 for both groups of resting-state EEG, considering the criteria of minimizing the cross-validation and maximizing the Krzanowski-Lai. We assign labels to the optimal prototype microstates, categorizing them into four classes: Class A, Class B, Class C, and Class D. Next, four prototype microstates undergo back fitting to all EEG recordings in the two groups. Each EEG time point is mapped to a specific prototype microstate based on its maximum spatial resemblance, thereby transforming the EEG into a microstate sequence. Finally, to enhance the fitting quality, the microstate sequence undergoes temporal smoothing and excludes microstates with durations shorter than 30 ms (Poulsen et al., 2018).

Following the completion of the microstate analysis, temporal features are computed for each of the four microstate classes in every patient. The global explained variance (GEV) is quantified as the percentage of similarity between the EEG and the assigned microstates, expressed as a percentage (%). The mean duration corresponds to the uninterrupted average duration of each microstate, measured in milliseconds (ms). The occurrence denotes the frequency at which each microstate occurs within a one-second interval, measured in hertz (Hz). The coverage represents the proportion of time that each microstate occupies throughout the recording, expressed as a percentage (%).

### 2.5. Microstate Lempel-Ziv complexity calculation

It can be observed that the microstate sequence represents the combination of four prototype microstates in the time domain, with each microstate having a short duration (see Figure 1). The length of the microstate sequence is slightly shorter than the preprocessed EEG data of the patients (due to the smoothing process, some microstates are discarded). The algorithm employed to compute the Lempel-Ziv complexity of the microstate time series in this study is consistent with previous research (Tait et al., 2020). Firstly, the microstate time sequence is transformed into a discrete sequence, where each time point corresponds to a distinct class within the four microstate topographies [A, B, C, D], thereby generating a string with the same number of sampling points as the original sequence. Then, the sequence is processed into a transitioning sequence (e.g., the sequence BBBCCAADDB is simplified to BCADB). Finally, the LZC value of the microstate sequence is calculated, which indicates the number of different subsequence patterns observed in the sequential analysis of the transformed sequence. Therefore, the values of microstate LZC indicate the number of distinct subsequences, reflecting the degree of repeatability and self-similarity during transitions of the four classes of microstates. Additionally, The algorithm determines that longer sequences have higher LZC values. As a result, when calculating the LZC, only the initial *N* substrings of the transitioning sequence are utilized. Since the length of our microstate sequence is below 60 s, we choose *N* = 300 to ensure that the minimum sequence length is greater than this value. For a comprehensive explanation of microstate LZC computation, please refer to Wegner et al. for detailed information (Von Wegner et al., 2023).

### 2.6. Statistical analysis

Group differences in patient age and post-stroke duration in weeks are evaluated through independent sample *t*-tests. The distribution of gender among patients is examined using the non-parametric chi-square test. To assess significant disparities in microstate temporal parameters between the PS-AW and PS-DOC groups, Welch’s *t*-test is employed. Moreover, DAR and microstate LZC comparisons between the two groups are conducted using both Welch’s *t*-test and the non-parametric Mann–Whitney *U* test. All statistical analyzes are performed using SPSS software (Version 25.0, IBM Inc., United States). The level of statistical significance is defined as a *value of p* less than 0.05 (or *p* < 0.01, *p* < 0.001) in this study. Additionally, microstate topography differences among microstate classes between the groups are assessed using a topographic analysis of variance (TANOVA) in the Ragu software (Koenig et al., 2011), employing 1,000 randomizations to establish a significance threshold of *p* < 0.05.

### 2.7. Classification of patients with PS-DOC

Thus far, we have obtained three types of group features, including the spectral feature DAR, the three microstate temporal features (mean duration, occurrence, and coverage) of four microstate classes, and the microstate LZC feature based on nonlinear measurement. Based on subsequent inter-group feature statistical analysis results, all three types of features demonstrate the differentiation between the two groups. Therefore, these features and their combinations are used as input for a SVM model with a radial basis function kernel to classify PS-DOC patients. The choice of radial basis function kernel SVM over other classifiers is attributed to its better scalability concerning the size of samples and features, as well as its ability to handle binary classification problem with multiple feature sets. The SVM model is implemented using the scikit-learn library in Python, with the hyperparameter *C* set to 1 to control the regularization degree. To accurately assess the performance of the model, 10-fold cross-validation is employed, and the average results are used to determine the values of sensitivity, specificity, accuracy, and the area under the curve (AUC) of receiver operating characteristic, which are four evaluation metrics.

## 3. Results

### 3.1. Electroencephalography microstate topographies

**Figure 2:**
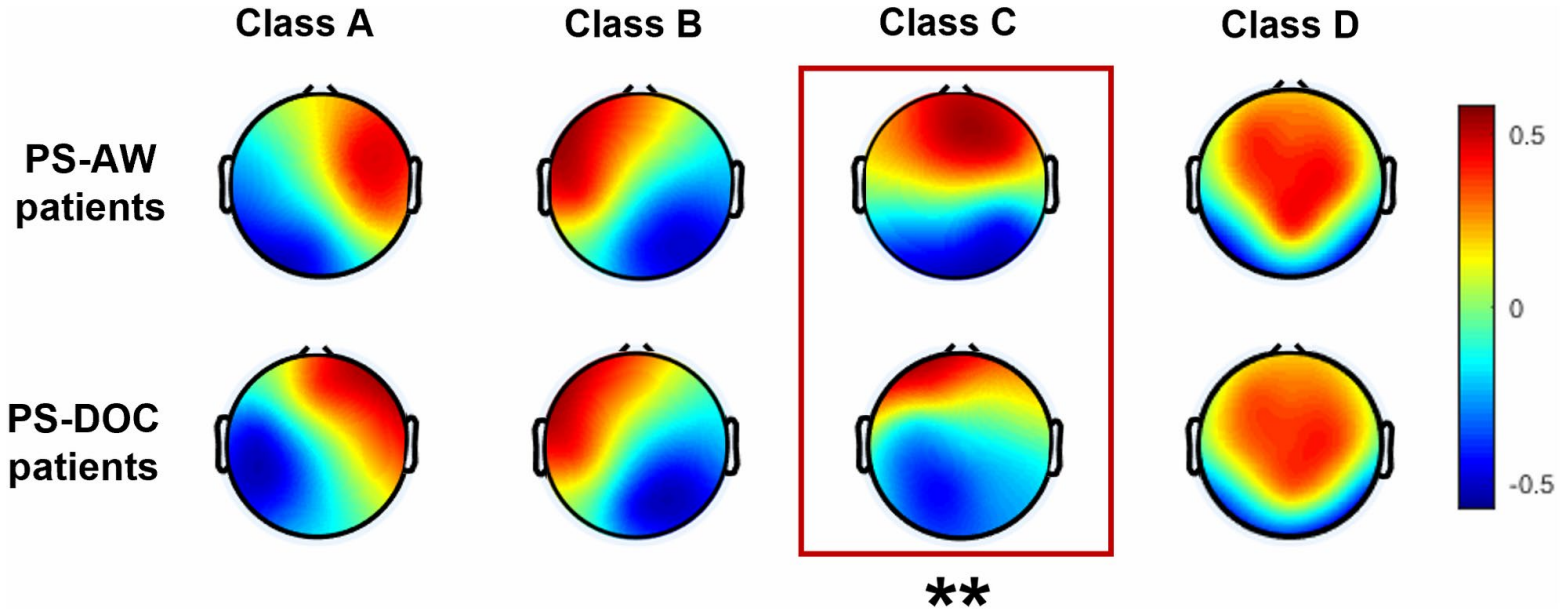
Four EEG microstate topographies for the PS-AW patient group (top) and PS-DOC patient group (bottom). The asterisks (**) indicate a significant difference in the microstate C between the two groups (*p* < 0.01) after TANOVA.

For the PS-AW group and the PS-DOC group, four EEG microstate topographies in each group are shown in Figure 2. Class A and Class B predominantly occupy the unilateral frontal region in a diagonal pattern, while Class C exhibits a vertical distribution. Class D, on the other hand, dominates the frontal-central area. These topographies are consistent with previous research (Zappasodi et al., 2017; Michel and Koenig, 2018). After the TANOVA, only the microstate topography of class C demonstrates a notable distinction (*p* = 0.0081). In addition, the topographies of the four microstates explain 75.18 and 73.79% of the GEV in the PS-AW and PS-DOC groups, respectively, indicating that these four microstate topographies can largely account for resting-state EEG. Furthermore, the difference in GEV between the two groups is not statistically significant (*t* = −1.345, *p* = 0.184).

### 3.2. Electroencephalography microstate temporal features

Next, Levene’s test reveals a violation of the assumption of homogeneity of variances for the temporal features of microstates between the PS-AW group and the PS-DOC group. Consequently, Welch’s *t*-test is conducted to compare the differences between the two groups. The mean values of mean duration, occurrence, and coverage for the four microstate classes in both groups, along with the *t*-test results, are presented in Table 2. The *t*-value in this context represents a standardized measure of the difference between sample means relative to the variability of the difference. A negative *t*-value indicates that the mean of the PS-AW group is smaller than the mean of the PS-DOC group, while a positive value indicates the opposite. Bold highlights in the table indicate statistically significant differences (*p* < 0.05). We observe that the temporal features of the microstate classes exhibit significant intergroup differences, except for the occurrence of microstate A and microstate C, and the coverage of microstate A.

**Table 2:** Comparison of EEG microstate temporal features, microstate LZC and DAR between PS-AW and PS-DOC patient groups.

		PS-AW (*n* = 28)		PS-DOC (*n* = 28)		*t-*value	Value of *p*
		Mean	SD	Mean	SD		
Mean duration	ClassA	83.642	11.781	92.487	10.543	−2.961	0.005**
	ClassB	68.250	3.544	76.805	7.492	−5.461	0.000***
	ClassC	81.019	4.104	90.156	5.158	−6.417	0.000***
	ClassD	87.737	6.127	84.581	4.889	2.131	0.038*
Occurrence	ClassA	3.548	0.591	3.376	0.340	1.324	0.193
	ClassB	2.412	0.400	2.709	0.279	−3.227	0.002**
	ClassC	3.118	0.205	3.129	0.350	−0.145	0.885
	ClassD	3.288	0.219	3.014	0.438	2.964	0.005**
Coverage	ClassA	30.953	7.599	33.881	6.337	−1.566	0.123
	ClassB	17.592	4.114	19.980	2.680	−2.574	0.013*
	ClassC	24.643	2.727	27.530	5.604	−2.451	0.019*
	ClassD	25.531	5.129	19.314	2.405	5.807	0.000***
LZC		112.02	6.577	104.139	5.114	5.006	0.000***
DAR		3.708	0.928	5.311	1.584	−4.046	0.000***

SD, standard deviation. Significant correlations are bolded after statistical analysis using Welch’s *t*-test. *p < 0.05; **p < 0.01; ***p < 0.001.

**Figure 3:**
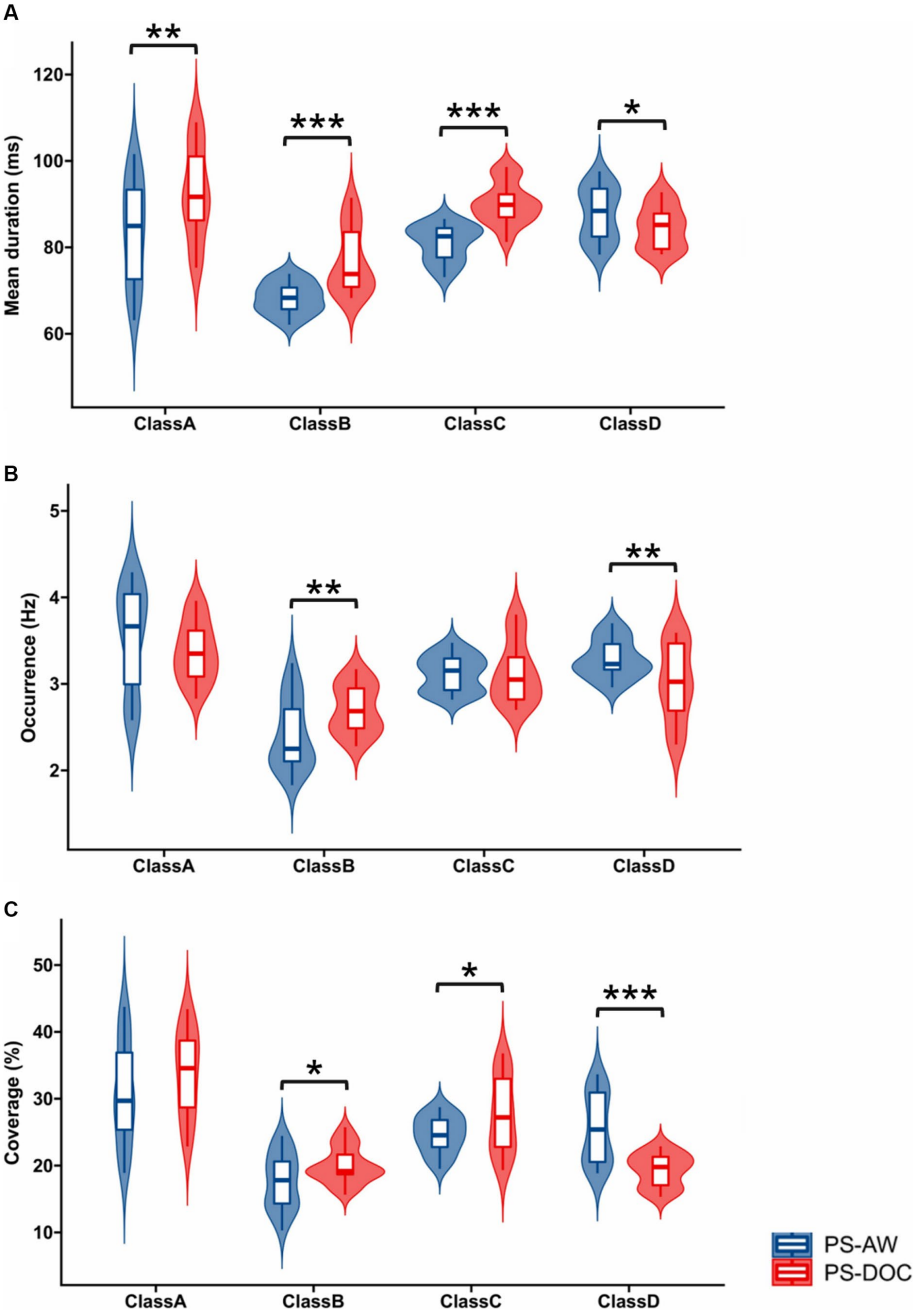
Inter-group statistics for temporal features of four microstate classes by Welch’s *t*-test. Violin plots and box plots depict the **(A)** Mean duration **(B)** Occurrence and **(C)** Coverage for each microstate class in the PS-AW and PS-DOC patient groups. *p < 0.05; **p < 0.01; ***p < 0.001.

Figure 3 provides violin plots and box plots for a visual representation of temporal features of microstates between the PS-AW group and the PS-DOC group. Regarding the horizontal comparison of the temporal features, all microstate classes, except for microstate D, show significant prolongation in mean duration for the PS-DOC group (*p* < 0.01). In terms of occurrence, significant differences are observed only for microstates B and D (*t* = −3.227, *p* < 0.01; *t* = 2.964, *p* < 0.01). As for coverage, microstate D exhibits a substantial decrease in the PS-DOC group (*p* < 0.001), while microstates B and C demonstrate an increase (*p* < 0.05). Shifting to the vertical comparison, microstates B and C consistently demonstrate enhancements across mean duration and coverage in the PS-DOC group, while microstate D consistently displays reductions. In other words, microstate B and microstate D consistently exhibit opposite patterns of change in temporal features between the two groups.

### 3.3. Delta/alpha ratio

**Figure 4:**
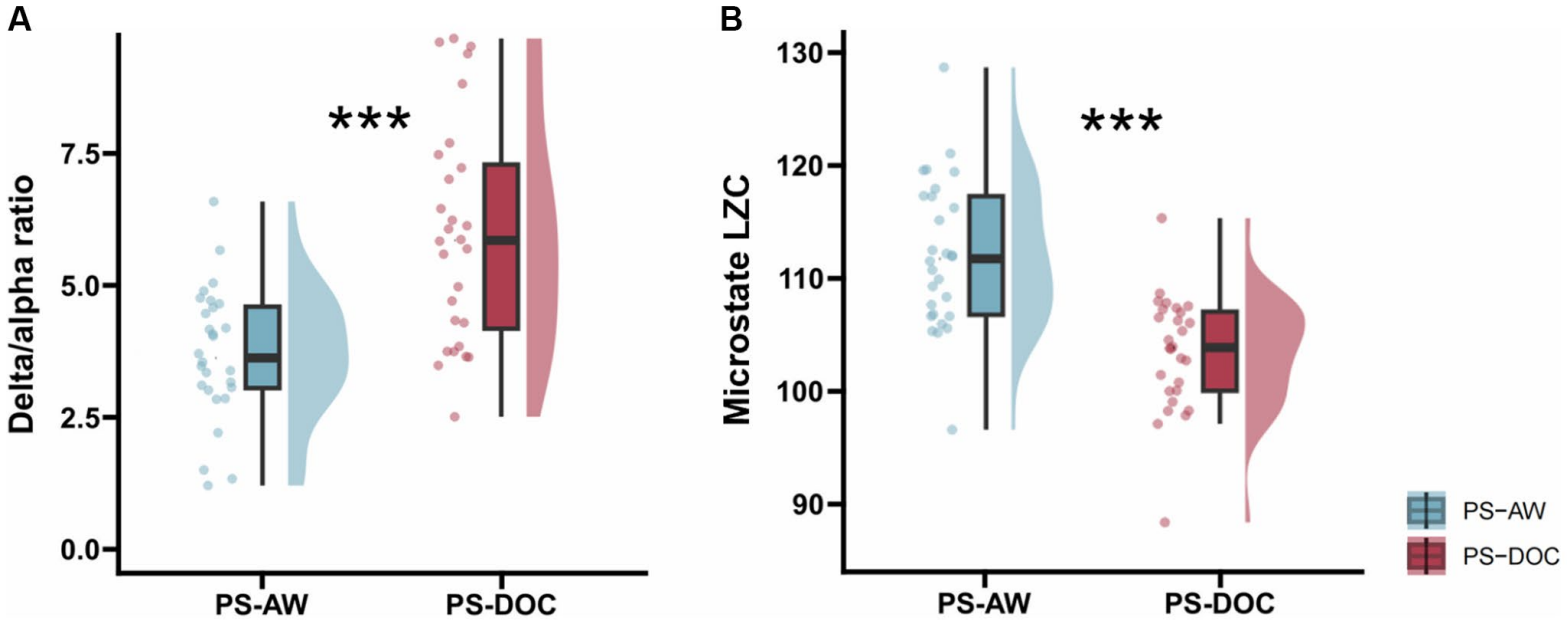
Inter-group statistics for DAR and microstate LZC by non-parametric Mann–Whitney *U* test. **(A)** DAR for the PS-AW and PS-DOC patient groups **(B)** Micostate LZC for the PS-AW and PS-DOC patient groups. ***p < 0.001.

In the spectral analysis, we compute the DAR of resting-state EEG for each patient in two groups, which is used to quantify the power spectral changes. Table 2 displays the results of intergroup comparisons using Welch’s *t*-test. The mean DAR for the PS-AW patient group is 3.708 (range: 1.26 to 5.98), while the mean DAR for the PS-DOC patient group is 5.311 (range: 2.48 to 9.76), and the DAR exhibits significant variation between the two groups (*t* = −4.046, *p* < 0.001). The results indicate that the average power in the delta frequency band is higher than that in the alpha frequency band for both patient groups, as the DAR ranges are not lower than 1. Furthermore, to provide comprehensive and reliable statistical inferences, a non-parametric Mann–Whitney *U* test is employed. As depicted in Figure 4A, the results likewise demonstrate that PS-DOC patients show a significant elevation of DAR compared to PS-AW patients (*U* = 185, *z* = −3.392, *p* < 0.001).

### 3.4. Microstate Lempel-Ziv complexity

For each patient’s microstate sequence, we calculate the LZC of their microstate transition sequence within a range of *N* = 300. The PS-DOC group has a mean microstate LZC value of 104.139, which is lower than the mean value of 112.02 in the PS-AW group. As depicted in Table 2 and Figure 4B, using Welch’s *t*-test and the non-parametric Mann–Whitney *U* test, we find a significant decrease in microstate LZC for the PS-DOC group (*t* = 5.006, *p* < 0.001; *U* = 185, *z* = −3.392, *p* < 0.001). The reduction of microstate LZC indicates that the four microstates of PS-DOC patients demonstrate higher repeatability and limited patterns of microstate transitions.

### 3.5. Classification

The results of inter-group statistical comparisons reveal that the EEG spectral feature DAR, along with the temporal features of microstates and the complexity feature microstate LZC disclose abnormal alterations in brain activity among patients with PS-DOC. We construct SVM models using different feature sets to assess whether the different combinations of these features could serve as potential effective biomarkers for PS-DOC. In terms of feature selection, we consider the temporal features (mean duration, occurrence, and coverage) of four microstate classes as Feature Set 1 (12 features in total). Combining DAR and microstate LZC with the temporal features of microstates respectively, we obtain Feature Set 2 and Feature Set 3 (both sets have 13 features). Finally, Feature Set 4 includes all three types of features (14 features in total). The principal component analysis is employed to reduce the dimensionality of these feature sets. Then, the four different feature sets are input into the radial basis function kernel SVM to classify PS-DOC patients, respectively. As shown in Table 3, when the three types of features are combined, the accuracy, sensitivity, and specificity are all above 89%. Specifically, the sensitivity is 92.86% (26/28 PS-DOC patients classified as PS-DOC by the model), indicating better classification performance compared to using these features individually or in other combinations.

**Table 3:** Classification performance comparison with 4 different feature sets.

Feature set	Accuracy	Sensitivity	Specificity	AUC	Feature number
Set1: Microstate temporal features	77.59%	78.57%	82.14%	0.831	12
Set2: Microstate temporal features + DAR	84.48%	89.29%	85.71%	0.915	13
Set3: Microstate temporal features + microstate LZC	81.03%	82.14%	89.29%	0.902	13
Set4: Microstate temporal features + DAR + microstate LZC	**91.07%**	**92.86%**	**89.29%**	**0.938**	14

Set4 shows the highest classification performance and is shown in bold.

## 4. Discussion

Over the past decade, the integration of electrocorticography, functional magnetic resonance imaging, and EEG source imaging techniques in the analysis of EEG microstates has revealed a close relationship between microstates and RSNs along with their corresponding cortical regions (Yuan et al., 2012; Khanna et al., 2015; Gschwind et al., 2016; Bréchet et al., 2019; Zoubi et al., 2020; Rajkumar et al., 2021; Mikutta et al., 2023), demonstrating their capacity to evaluate the large-scale electrical activity in cortical networks (Tarailis et al., 2023). In this study, both the PS-AW group and the PS-DOC group exhibit four typical microstate topographies, with noticeable differences observed in the topological structure of microstate C. Regarding the source localization and specific association of microstate C with RSNs, a consensus has not yet been reached. Previous studies suggested an association between microstate C and the salience network (Britz et al., 2010), while recent research indicated its potential origins from the posterior cingulate cortex and precuneus (Custo et al., 2017; Zanesco et al., 2021), regions known for their critical role in the default mode network (DMN) (Fransson and Marrelec, 2008; Hagmann et al., 2008). An investigation on the consciousness level of brain-injured patients demonstrated a significant peak in DMN connectivity within the posterior cingulate cortex, highlighting the strong correlation between activity in this region and patients’ consciousness levels (Cavanna and Trimble, 2006; Vanhaudenhuyse et al., 2010). Croce et al. found that transcranial magnetic stimulation to the two pivotal regions of the DMN significantly altered the microstate C topography (Croce et al., 2018), suggesting the potential of microstates in elucidating local inhibition within brain regions. These findings imply that the decreased consciousness level in patients with stroke-induced brain damage may be associated with the inhibition of key areas within the DMN. This inhibition is likely to result in alterations in the microstate C topography, reflecting changes in neural activity and connectivity.

Furthermore, we observe a noticeable increase in the mean duration and coverage of microstate C in the PS-DOC patient group, which is in line with recent research (Toplutaş et al., 2023), although the DOC patients in their study cohort were of different aetiologies and their control group consisted of healthy subjects. These findings imply that the temporal features of microstate C may not be sufficiently sensitive to differentiate DOC patients caused by stroke and those with other etiologies, however, it still holds potential as a biomarker for DOC diagnosis. Another study reported a substantial reduction in the mean duration and coverage of microstate C in improving DOC patients during the consciousness recovery process following high-definition transcranial direct current stimulation (Guo et al., 2022), which further supports this viewpoint.

Regarding microstate D, we find a noticeable decrease in the mean duration, coverage, and occurrence in the PS-DOC group, which is opposite to the increased temporal parameters of microstates B and C. Microstate D is negatively correlated with the BOLD signals of the frontal and parietal lobes’ right posterior dorsal regions, which correspond to the dorsal attention network (DAN) activity (Britz et al., 2010; Michel and Koenig, 2018). Indeed, the opposite behavior of microstates C and D has been found in previous cognitive manipulation tasks, where the temporal measures of microstate D showed a significant augmentation in tasks involving the DAN, while the metrics of microstate C decreased (Seitzman et al., 2017). These research findings imply dysfunction of the DAN network in PS-DOC patients. Moreover, microstate D is suggested to represent certain aspects of attention and focus switching, and its temporal parameters show a decrease in response to reduced alpha power (Croce et al., 2020). The increase in DAR caused by the decreased alpha power observed in the PS-DOC group appears to explain the reduced time features of microstate D, indicating a dysregulation of attention in PS-DOC patients compared to PS-AW patients.

Moreover, The PS-DOC patient group shows an extended time parameter for microstate B, consistent with the results of Toplutaş et al. (2023) who found a remarkable increase in the mean duration and coverage of microstate B in DOC patients. Comparable results have been found in other neurological disorders, such as increased microstate B occurrence and coverage in migraine patients (Li et al., 2022), and higher mean duration, occurrence, and coverage of microstate B in adolescent depression patients (Zhao et al., 2022). Furthermore, microstate B has a negative correlation with bilateral occipital lobe BOLD activation (Michel and Koenig, 2018), and it has a 36% overlap with the DAN, followed by the visual network and the ventral attention network (36 and 31%, respectively; Zoubi et al., 2020). This suggests a relationship between the attenuated indicators of microstate D and the enhanced indicators of microstate B in PS-DOC patients, but further explanation is needed using more precise brain imaging techniques. Lastly, similar to microstates B and C, there is a significant increase in the mean duration of microstate A in the PS-DOC patient group. Guo et al. (2022) found a negative correlation between the CRS-R scores and the mean duration of microstate A in patients with DOC, which indicates that the mean duration of microstate A decreases gradually during the treatment process of consciousness recovery. Similarly, a longer mean duration of microstates in patients with DOC was associated with lower brain dynamical activity (Zhang et al., 2023). Moreover, the sleep or anesthesia state showed an increased mean duration of microstates in comparison to the awake state (Bréchet and Michel, 2022). In our study, the PS-AW group exhibits a shorter mean duration of microstates compared to the PS-DOC group, indicating that PS-DOC patients require more time for brain information transmission and processing under the same microstates and RSNs, which may affect the patient’s consciousness level.

The quantitative analysis of the frequency domain on resting-state EEG is indispensable. As mentioned earlier, several investigations have reported augmented delta-wave activity coupled with reduced alpha-wave activity in both stroke patients (Schleiger et al., 2014) and DOC patients following brain injury (Varotto et al., 2014). In this study, our results show an appreciable increase of DAR in the PS-DOC patient group, consistent with previous reports in brain injury and DOC patients. DAR correlated strongly negatively with functional recovery in acquired brain injury (Leon-Carrion et al., 2009), and Finnigan et al. found that a DAR ratio of 3.7 accurately distinguished ischemic stroke patients (Finnigan et al., 2016). Furthermore, Lechinger et al. (2013) found a high correlation between spectral peak frequency and CRS-R scores, and delta wave power exhibited a significant negative correlation with CRS-R scores (Bareham et al., 2018). These findings provide robust evidence for the increased DAR in PS-DOC patients and further emphasize the importance of using DAR as a quantitative EEG measure for DOC diagnosis and assessment.

Furthermore, there is a theoretical proposition that neural complexity is one of the important foundations of consciousness formation (Tononi, 2005, 2008), and the brain exhibits higher complexity in information integration and processing in states of higher consciousness levels (Dehaene and Changeux, 2011). These viewpoints have been validated in studies of EEG complexity in DOC patients, such as significantly lower complexity measured by LZC values in DOC patients (Wu et al., 2011), and a non-monotonic increase in LZC during the consciousness recovery process (Lei et al., 2022). In recent years, researchers have utilized the LZC feature of microstate time series to study the neurodynamics of patients with neurological disorders at a smaller time scale (Tait et al., 2020; Artoni et al., 2022). In our study, we apply the microstate LZC to DOC patients for the first time, and the results display lower microstate LZC values in the PS-DOC patient group, indicating a tendency for more repetitive and slower transitions of the microstates in the PS-DOC group, which may reflect a decrease in complexity during information integration and distributed processing. Additionally, a recent study found lower EEG LZC and permutation LZC to be associated with residual consciousness in DOC patients (Liu et al., 2023), which supports our results.

In recent years, significant progress has been made in the classification methods for DOC. This progress can largely be attributed to the application of electrophysiological methods in measuring brain neural function with the need to supplement the criteria for DOC classification with information from EEG (Bayne et al., 2017). Recent research proposed a multiple-scale convolutional few-shot learning network that can extract more information from EEG and applied it to evaluate DOC patients with residual consciousness in a P300-based brain-computer interface (BCIs) (Pan et al., 2023). Although there have been preliminary results in the assistance of diagnosing DOC patients using BCIs, BCI tasks often require active cooperation from patients, which remains a significant challenge for DOC patients (Galiotta et al., 2022). Another research attempted to maximally utilize functional connectivity information in brain networks to evaluate DOC, using convolutional neural networks and network reconfiguration techniques to classify patients with different consciousness states, achieving a classification accuracy of 87.2% (Cai et al., 2023). However, a recent study demonstrated that compared to convolutional neural networks or deep learning algorithms, SVM classifier showed stability in classifying various EEG signal preprocessing patterns for tasks such as DOC classification and had better interpretability, making them more suitable for doctors and patients to adopt (Rosenfelder et al., 2023). Our research explores the potential value of EEG microstates in DOC classification, which has not been explored in previous studies according to the available literature. We employ an SVM classifier to identify DOC patients among stroke patients using only the temporal features of the four microstate classes (accuracy = 77.59%), and obtain accuracies of 84.48 and 81.03% when incorporating the spectral feature DAR and the microstate complexity feature LZC, respectively. When combining DAR and microstates’ temporal and LZC features together, the highest accuracy and sensitivity are achieved (accuracy = 91.07%, sensitivity = 92.86%). The microstate temporal sequences and topographies have higher temporal and spatial resolution compared to EEG sequences. The features extracted from microstates can be complemented with frequency-domain information to improve the performance of classification, and higher sensitivity in classification is crucial for diagnosing patients with PS-DOC in a clinical setting.

Despite providing potential electrophysiological biomarkers for the post-stroke disorder of consciousness, this study has several limitations to consider. Firstly, the study only includes DOC patients caused by subacute ischemic stroke, which, although avoiding potential influences from other etiologies, still has high heterogeneity among ischemic stroke patients, such as lesion location and size, stroke severity, etc. These factors need to be considered in future studies for their impact on group analysis. Secondly, due to missing clinical scale scores in some DOC patients in the study cohort, the correlation analysis between different features and clinical scales is not conducted. Therefore, future studies need to supplement the correlation information between the features and scales. Finally, this study highlights the potential role of these features in the diagnosis and assessment of PS-DOC. However, to delve deeper into the observed uniqueness, future research should include longitudinal studies to investigate the effectiveness of these indicators in predicting stroke patient prognosis.

## 5. Conclusion

In this study, we conduct EEG microstate analysis on patients with DOC resulting from a single etiology, specifically ischemic stroke. Our findings reveal distinct differences in the topography of microstate C between PS-DOC patients and PS-AW patients, indicating localized suppression of DMN activity in PS-DOC. Microstates B and C exhibit significant increases in temporal features in the PS-DOC group compared to the PS-AW group, while microstate D shows the opposite pattern. These novel microstate findings provide valuable insights and additional information on abnormal RSN changes in DOC. Moreover, integrating EEG frequency domain analysis and nonlinear analysis of microstates, we observe notable increases in the DAR and reductions in the microstate LZC within the PS-DOC group. Lastly, utilizing these distinctive features as a comprehensive set, we achieve high accuracy in classifying PS-DOC patients (Accuracy = 91.07%). Our findings emphasize the potential of EEG microstates as promising electrophysiological biomarkers for the assessment of DOC among stroke patients, with further enhancements possible through the integration of spectral features.

## Data availability statement

The datasets presented in this article are not readily available because individual patient data cannot be made available under Chinese law because we did not obtain patient approval for sharing individual patient data, even in the form of electroencephalogram (EEG) data. Requests to access the datasets should be directed to lifenglian@tyut.edu.cn.

## Ethics statement

The studies involving humans were approved by the Ethics Committee of Shanxi Provincial People’s Hospital. The studies were conducted in accordance with the local legislation and institutional requirements. The ethics committee/institutional review board waived the requirement of written informed consent for participation from the participants or the participants’ legal guardians/next of kin because written informed consent for participation was not required for this retrospective study in accordance with the national legislation and the institutional requirements.

## Author contributions

FY: Conceptualization, Methodology, Formal analysis, Investigation, Software, Writing—original draft, Writing—review and editing. YG: Methodology, Formal analysis, Writing—original draft, Writing—review and editing. FL: Conceptualization, Methodology, Formal analysis, Data collection, Writing–original draft, Writing–review and editing, Funding acquisition, Supervision. XZ: Data collection, Funding acquisition, Supervision. FH: Data collection, Investigation, Software. WJ: Data collection, Investigation, Funding acquisition. XL: Investigation, Writing–review and editing.

## Funding

The author(s) declare financial support was received for the research, authorship, and/or publication of this article. The study leading to these results received funding from the National Natural Science Foundation of China, the Youth Fund of Shanxi Health Commission, Provincial Special Supporting Fund Project of Shanxi Provincial People’s Hospital, under Grant Agreement Nos. 62171307, 201301029, and sj20019007.

## Conflict of interest

The authors declare that the research was conducted in the absence of any commercial or financial relationships that could be construed as a potential conflict of interest.

## Publisher’s note

All claims expressed in this article are solely those of the authors and do not necessarily represent those of their affiliated organizations, or those of the publisher, the editors and the reviewers. Any product that may be evaluated in this article, or claim that may be made by its manufacturer, is not guaranteed or endorsed by the publisher.
